# Dabigatran pharmacokinetic-pharmacodynamic in sheep: Informing dose for anticoagulation during cardiopulmonary bypass

**DOI:** 10.1177/02676591231226291

**Published:** 2024-01-03

**Authors:** Michael P Eaton, Sergiy M Nadtochiy, Tatsiana Stefanos, Brian J Anderson

**Affiliations:** 1 6923 University of Rochester Medical Center, New York, NY, USA; 2 Department Anesthesiology, 62710 University of Auckland, Auckland, New Zealand

**Keywords:** Dabigatran, coagulation, cardiopulmonary bypass, pharmacokinetics, pharmacokinetics, idarucizumab

## Abstract

**Background:**

The effect of the anticoagulant, dabigatran, and its antagonist, idarucizumab, on coagulation remains poorly quantified. There are few pharmacokinetic-pharmacodynamic data available to determine dabigatran dose in humans or animals undergoing cardiopulmonary bypass.

**Methods:**

Five sheep were given intravenous dabigatran 4 mg/kg. Blood samples were collected for thromboelastometric reaction time (R-time) and drug assay at 5, 15, 30, 60, 120, 240, 480 min, and 24 h. Plasma dabigatran concentrations and R-times were analyzed using an integrated pharmacokinetic-pharmacodynamic model using non-linear mixed effects. The impact of idarucizumab 15 mg/kg administered 120 min after dabigatran 4 mg/kg and its effect on R-time was observed.

**Results:**

A 2-compartment model described dabigatran pharmacokinetics with a clearance (CL 0.0453 L/min/70 kg), intercompartment clearance (Q 0.268 L/min/70 kg), central volume of distribution (V1 2.94 L/70 kg), peripheral volume of distribution (V2 9.51 L/70 kg). The effect compartment model estimates for a sigmoid E_MAX_ model using Reaction time had an effect site concentration (Ce_50_ 64.2 mg/L) eliciting half of the maximal effect (E_MAX_ 180 min). The plasma-effect compartment equilibration half time (T_1/2_keo) was 1.04 min. Idarucizumab 15 mg/kg reduced R-time by approximately 5 min.

**Conclusions:**

Dabigatran reversibly binds to the active site on the thrombin molecule, preventing activation of coagulation factors. The pharmacologic target concentration strategy uses pharmacokinetic-pharmacodynamic information to inform dose. A loading dose of dabigatran 0.25 mg/kg followed by a maintenance infusion of dabigatran 0.0175 mg/kg/min for 30 min and a subsequent infusion dabigatran 0.0075 mg/kg/min achieves a steady state target concentration of 5 mg/L in a sheep model.

## Introduction

Dabigatran is an anticoagulant drug that reversibly binds to the active site of the thrombin molecule; it is classified as a direct thrombin inhibitor. It also reduces thrombin-mediated inhibition of fibrinolysis. The USA Food and Drug Administration (FDA) has approved oral dabigatran for the management of adult humans with thromboembolic disease and stroke prevention in those with atrial fibrillation. It is also available for the treatment of children ages 3 months to 12 years with venous thromboembolism directly after receiving injectable anticoagulants for at least 5 days.^1^ Dabigatran has a volume of distribution of 50–70 L in humans with an elimination half-life of 12–17 h. When administered orally the relative bioavailability (F) is only 3%–7%. Adult human apparent clearance estimates (CL/F ∼ 4 L/min)^2,3^ are influenced by renal function,^4^ although hepatic glucuronide metabolism to acyl metabolites also contributes.^2^ Dabigatran anticoagulant effect can be reversed using the monoclonal antibody, idarucizumab. Intravenous idarucizumab immediately decreases unbound dabigatran concentration.^5^ A linear correlation was observed between unbound dabigatran and diluted thrombin time and ecarin clotting time (ECT).^5^ Dabigatran is contraindicated in patients with active pathological bleeding and its major adverse effect is gastrointestinal bleeding.^6^

Other direct thrombin inhibitors (e.g., bivalirudin, hirudin, argatropan) have been successfully used as an alternative to heparin anticoagulation for patients undergoing cardiopulmonary bypass.^7–9^ Dabigatran also has promise as a direct thrombin inhibitor for cardiopulmonary bypass,^10,11^ in part because the effects of dabigatran can be rapidly and completely reversed with the monoclonal antibody, idarucizumab.^12^ Preliminary results suggest that effects of dabigatran concentration on diluted thrombin time (dTT), ecarin clotting time (ECT) and activated partial thromboplastin time (aPTT) are largely comparable between adults and children.^13^

Unfractionated heparin is the most commonly used anticoagulant in both cardiopulmonary and extracorporeal circuits. Concerns about heparin induced thrombocytopaenia has spurred examination of direct thrombin inhibitors.^14^ However, bivalirudin and argatropan have no reversal agent available. Dabigatran is a potential alternative because effect is reversable. Investigation using animal models is a first step used to guide investigation in humans. We investigated the dabigatran pharmacokinetic-pharmacodynamic relationship in sheep using a delayed effect compartment model.^15,16^ The effect measure of reaction time (R-time) was used as a convenient point-of-care whole blood test reflecting inhibition of the tissue factor pathway. Our intent was to use the target concentration strategy^17,18^ to determine a dabigatran dose that could be used for anticoagulant investigation of sheep undergoing cardiopulmonary bypass. This strategy involves identification of a target dabigatran concentration for cardiopulmonary bypass use in sheep. A dose to achieve and maintain this target concentration can then be investigated using compartment models describing drug disposition in a cardiopulmonary bypass circuit.

## Methods

### Animals and materials

Five sheep were available for study. Demographic data are shown in Table 1. Jugular or cephalic veins were cannulated with 24 g intravenous catheters (Jelco ®, ICU Medical, Inc., San Clemente, CA, USA). Subsequent cannulation (e.g., peripheral arterial line placed in the auricular artery) and the dabigatran pharmacokinetic-pharmacodynamic (PKPD) study were performed under general anaesthesia. Anaesthesia was induced using intravenous ketamine 4 mg/kg plus midazolam 0.4 mg/kg. Endotracheal intubation was performed and anaesthesia maintained using inhalational isoflurane 1%–4%. At the conclusion of the study period sheep had intravenous and arterial cannulas removed and hemostasis from cannulation sites achieved with pressure.

**Table 1.** Demographic data and dabigatran injectate volume of sheep who underwent PKPD analysis.

Sheep identification	Weight (kg)	Age (months)	Sex	Dabigatran injectate volume (mL)
1	28.7	6	Female	29.8
2	33.9	6	Female	35.3
3	33.8	6	Female	35.2
4	33.9	6	Female	35.3
5	41.8	6	Female	43.5

Dabigatran 4 mg/kg IV was injected over 1 min immediately after the baseline (time 0) draw and flushed with 3 mL of normal saline. Blood samples for dabigatran assay and R time were taken at 5, 15, 30, 60, 90, and 120 min. Idarucizumab 15 mg/kg IV was given over 30 s immediately after the 120 min of dabigatran injection time, and additional blood samples were collected at the subsequent times of 5, 15, 30, 60, 120, 240, 480 min, and 24 h. All experimental protocols were approved by the Association for Assessment and Accreditation of Laboratory Animal Care-accredited University of Rochester Committee on Animal Resources.

Dabigatran (4 mg/kg) was injected through the venous cannula manually over 60 s. Samples (2 mL) were collected into citrate tubes from the arterial cannula at baseline (prior to injection) and at 5, 15, 30, 60, 90 and 120 min while the sheep were under anesthesia. Idarucizumab 15 mg/kg was injected through the venous cannula manually over 30 s, and blood samples were collected at 5, 15, 30, 60 min while the sheep were under anesthesia. Then sheep were recovered from anesthesia and moved back to their cages with food and water ad libitum. The remaining late blood samples at 120, 240, 480 min and 24 h after idarucizumab administration were collected. The total amount of collected blood was less than 7.5% (4.5 mL/kg body weight). At the conclusion of the study period sheep had intravenous and arterial cannulas removed and hemostasis from cannulation sites achieved with pressure. Sheep were monitored for 24 h for adverse reactions. Markers of hepatic and renal injury including alanine aminotransferase (ALT), aspartate aminotransferase (AST), and creatinine concentration were measured at baseline and at 24 h.

Reaction time (R-time) was measured using a Thromboelastograph Analyzer 5000 (Haemoscope Corp.). Plasma dabigatran concentrations were measured using liquid chromatography/mass spectrometry (LC-MS/MS) with a Dionex Ultimate 3000 UHPLC coupled to a Q Exactive Plus mass spectrometer (Thermo Scientific).

### Dabigatran solutions

An intravenous formulation was required for study. One milligram of Dabigatran (Clearsynth, Ontario, Canada) was dissolved in 60 µL of 0.075 M HCl, and then the solution was added into 0.2 mL of 20% N,N-dimethylacetamide. The dabigatran injectate concentration was 3846 ug/mL. Dabigatran volume injected intravenously over 1 min is shown in Table 1.

Reaction time (R), and dabigatran concentration were measured in all blood samples. One milliliter of blood was centrifuged at 370 g for 15 min to obtain plasma dabigatran concentrations measured using liquid chromatography/mass spectrometry (LC-MS/MS) with a Dionex Ultimate 3000 UHPLC coupled to a Q Exactive Plus mass spectrometer (Thermo Scientific, San Jose, CA).

### Dabigatran pharmacokinetic-pharmacodynamics

Population parameter estimates were obtained using nonlinear mixed effects models (NONMEM 7.5, ICON Development Solutions, MD, USA). These models account for population parameter variability (between subjects) and residual variability (random effects) as well as parameter differences predicted by covariate (fixed) effects. Population parameter variability was described using exponential models, which is equivalent to assuming a log-normal distribution and avoids biologically inappropriate parameter values of zero or less. Residual unidentified variability (RUV) was modeled using both proportional (RUV_PROP_) and additive residual (RUV_ADD_) errors. The ADVAN6 subroutine was used to solve differential equations. NM-TRAN code is available in supplementary material (Supplementary NM-TRAN Code). A sequential PPPD method was used for final pharmacodynamic parameter estimates.^19^ Convergence criterion was three significant digits.

#### Pharmacokinetics

**Figure 1.**
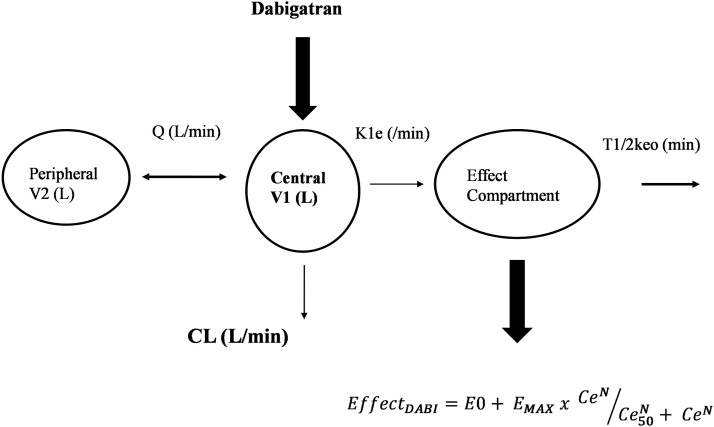
A diagram representing the pharmacokinetic effect compartment model. Drug is delivered into a central compartment (V1) that distributes to a peripheral compartment (V2) linked by an intercompartment clearance (Q). A rate constant (k1e) links the central compartment to an effect compartment. The rate constant k1e is the same as keo when the system is at equilibrium.

A two-compartment (central and peripheral) pharmacokinetic model was used to fit data. The model was parameterized in terms of clearance (CL), between compartment clearance (Q), central volume (V1) and peripheral volume of distribution (V2). An additional effect compartment was linked to the central compartment by a rate constant (keo). That constant was expressed as a half-time (T_1/2_keo = Ln(2)/keo), demonstrated in Figure 1. The pharmacokinetic parameter values were standardized for a body weight of 70 kg using allometric models.^20^ This standardization allows comparison of sheep parameter estimates with those reported for human adults^21^:

Pi=PSTD×(WiWSTD)EXP

where P_i_ is the parameter of the i^th^ subject, W_i_ is the weight of the i^th^ subject and P_STD_ is the parameter of standard weight W_STD_ of 70 kg. The EXP exponent was 0.75 for clearance and 1 for distribution volumes.^22^

#### Pharmacodynamics

A sigmoidal E_MAX_ model was used to describe thromboelastogram reaction time (R). The reaction time (R-time) represents the time until initial fibrin formation after mechanical stress and reflects the ability to generate thrombin. Population parameter estimates were estimated using an effect compartment model, a model valid for situations where there is an apparent temporal displacement between plasma concentration (Cp) and response e.g., neuromuscular blocking drugs.^15^ A rate constant (keo, T_1/2_keo = Ln(2)/keo) links plasma concentration with effect site concentration (Ce).

EffectDABI=E0+EMAX x CeNCe50N+CeN

The parameter E0 is the baseline measure (e.g., ACT 100, R 0.4 min), E_MAX_ is the maximum drug effect, Ce_50_ is the effect site concentration eliciting half of E_MAX_ and N is the Hill coefficient describing the steepness of the concentration–response curve^15^

The impact of idarucizumab was modelled by a unit bolus input into a fourth compartment with first order elimination described by a rate constant (K_IDA_). Input duration (DUR_IDA_) was estimated as a parameter. The vasopressors concentration (C_IDA_) was assumed to directly decrease R time and this relationship was described using a slope constant (SLOPE_IDA_). Observed R time was the sum of Dabigatran (EFFECT_DABI_) and idarucizumab effects (EFFECT_IDA_).

#### Quality of fit

Model selection required an improvement in the NONMEM objective function (OBJ) between nested models, equating to a reduction >3.84 based on a Chi square distribution (α < 0.05). A visual predictive check (VPC), was used to evaluate how well the model predicted the distribution of observed dabigatran concentrations or coagulation measures (R).

## Results

### Pharmacokinetics

**Figure 2.**
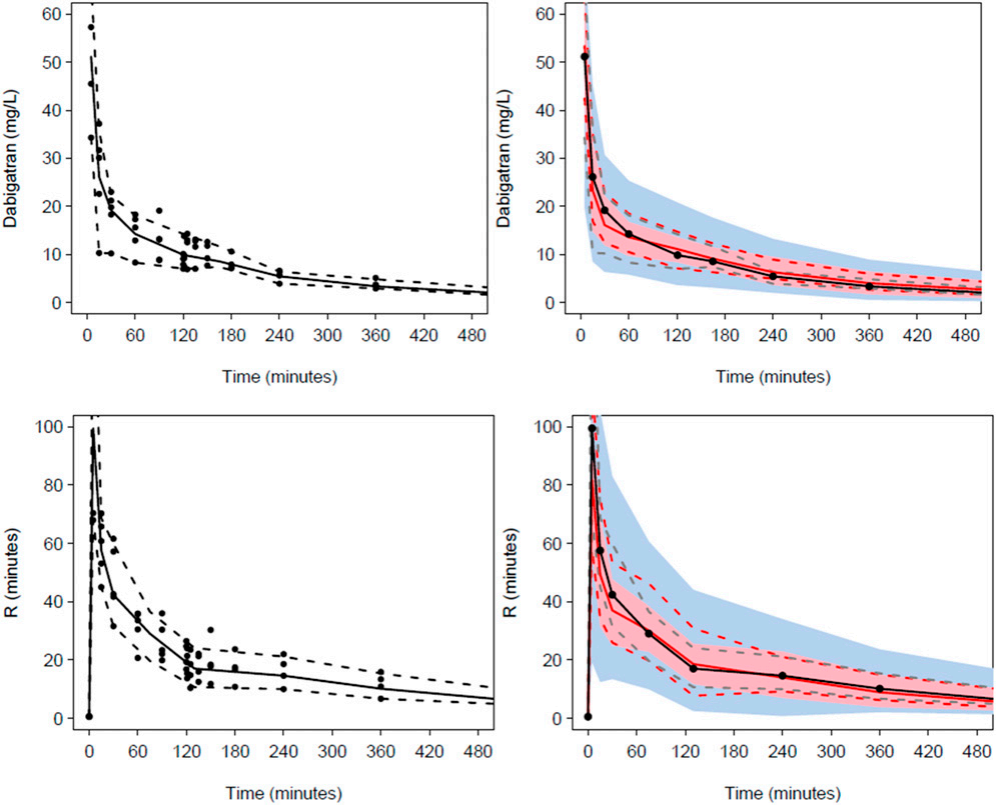
Visual predictive checks (VPC) Plots show median (solid) and 90% intervals (dashed lines). The left hand plot shows all prediction corrected observed dabigatran concentrations or effect (R-time). The right hand plot shows prediction corrected percentiles (10%, 50%, and 90%) for observations (grey dashed lines) and predictions (red dashed lines) with 95% confidence intervals for prediction percentiles (median, pink shading; 5th and 95th blue shading). The upper panel displays the VPC for the pharmacokinetic analysis. The lower panel shows the VPC for the pharmacodynamic reaction time (R) response.

Pharmacokinetic population parameter estimates for a sheep weight of 70 kg are shown in Table 2. Parameter estimates are scaled to a typical human weight of 70 kg for convenience of comparing characteristics between species. This does not change the relationship between size and parameters; it simply changes the scale of the parameter.^21^ The visual predictive check (VPC) is shown in Figure 2. Markers of hepatic and renal injury were unchanged at 24 h after dabigatran administration.

**Table 2.** Standardized dabigatran population pharmacokinetic parameter estimates. BSV is the between subject parameter variability, SE is the standard error, CI is the confidence interval.

Parameter	Estimate	%BSV	95% CI
CL_STD_ (L/min/70 kg)	0.0453	21.3	0.02–0.053
V1_STD_ (L/70 kg)	2.94	7.4	2.27–3.14
Q_STD_ (L/min/70 kg)	0.268	51.4	0.175–0.400
V2_STD_ (L/70 kg)	9.51	39.6	7.07–14.7
RUV_ADD_ (mg/L)	1.32	—	0.79–1.49
RUV_PROP_ (%)	0.47	—	0.42–0.49

### Pharmacodynamics

Pharmacodynamic population parameter estimates for R- times are shown in Table 3. The visual predictive check (VPC) plots for the effect of dabigatran on reaction time (Figure 2) confirmed the adequacy of model predictions with little apparent deviations between model and data. The 90% confidence interval and median for observed data lies within the predicted intervals were obtained by simulation.

**Table 3.** Pharmacodynamic population parameter estimates for reaction times (R).

Parameter	Estimate	BSV%	95%CI
Effect compartment model			
E0 (min)	0.681	15.1	0.547, 0.796
Emax (min)	180 FIX	—	—
Ce_50_ (mg/L)	64.2	19.9	56.6, 71.92
N	1 FIX	—	—
T_1/2_keo (min)	1.04	—	0.936, 2.14
K_IDA_	0.0218	105.4	0.009–0.075
DUR_IDA_	0.0751	—	—
SLOPE_IDA_	8.24	—	6.26, 12,45
RUV_ADD_ (min)	0.149	—	0.002-0.231
RUV_PROP_ (%)	37.0	—	34.3, 46.9

### Impact of idarucizumab on reaction time

The use of idarucizumab 15 mg/kg administered 120 min after dabigatran 4 mg/kg reduced reaction time by approximately 5 min over the 5 min after administration. This effect is demonstrated in Supplementary Figure S1 for sheep subject #4.

**Figure 3.**
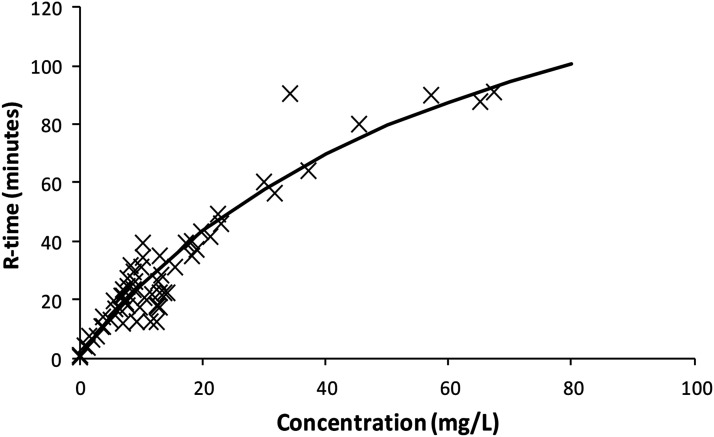
An Emax model was used to describe the relationship between dabigatran effect site concentration and R-time. The symbols (x) are individual Bayesian predictions. The solid line demonstrates population prediction. An R time of 13 min (Normal: 4–8 min) was considered a suitable target effect. The effect site concentration correlates with a target concentration of 5 mg/L.

### Target concentration strategy

The goal of treatment is the target effect. The target concentration strategy is useful for determining the clinical dose through an understanding of pharmacokinetics and pharmacodynamics.^17^ The concentration-response relationship (Figure 3), determined in the pharmacodynamic analysis was used to predict a target concentration of 5 mg/L. Pharmacokinetic parameters were used to determine dose that would achieve this target concentration in sheep supported using cardiopulmonary bypass.

**Figure 4.**
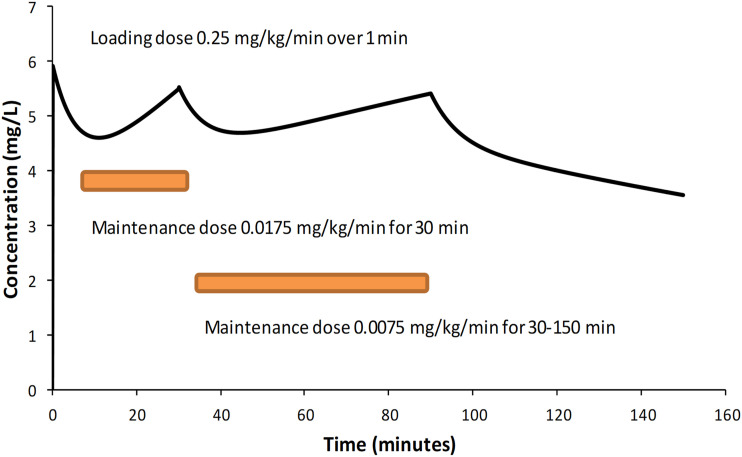
Simulation of dabigatran concentration using a loading dose of dabigatran 0.25 mg/kg followed by a maintenance infusion of dabigatran 0.0175 mg/kg/min for 30 min and a subsequent infusion dabigatran 0.0075 mg/kg/min for 30–90 min achieved a steady state target concentration of 5 mg/L.

### Determination of dabigatran dose

A simple pharmacokinetic model was used to determine dabigatran disposition in sheep during cardiopulmonary bypass (Supplementary Figure S2). Pharmacokinetic Parameter estimates (Table 2) were used to simulate time concentration profiles using differential equations in Berkeley Madonna™ modeling and simulation software (Robert Macey and George Oster of the University of California Berkeley, USA).

Differential equations used were:

d/dt (central) = drug rate in + Q2*C2 + Q3*C3*- (CL+Q2+Q3)*C1

d/dt (peripheral) = Q2*(C1 - C2)

d/dt (CPB) = C1*Q2-C3*Q3

where C1 is concentration in the central compartment, C2 is concentration in the peripheral compartment, and C3 that concentration in the cardiopulmonary bypass (CPB) circuit; Q2 is the intercompartment clearance and Q3 the pump flow, parameterized as intercompartment clearance Q3.

Loading doses and maintenance infusions over 90 min that achieve steady-state concentrations of 5 mg/mL are shown in Figure 4. These infusion rates assume that the CPB circuitry is preloaded with a dabigatran to achieve the target concentration of 5 mg/L. That dose is determined by the volume of the CPB circuit (V3).

Dabigatran Dose=target concentration (mgL) x V3 (L)

A loading dose of dabigatran 0.25 mg/kg followed by a maintenance infusion of dabigatran 0.0175 mg/kg/min for 30 min and a subsequent infusion dabigatran 0.0075 mg/kg/min achieved a steady state target concentration of 5 mg/L.

## Discussion

We present an example of use of the target concentration strategy to determine dose for cardiopulmonary bypass investigation of dabigatran in sheep. This methodology is commonly used for clinical drug development and dose determination.^17,18^ The goal of any pharmacological treatment is the target effect. A pharmacodynamic model for dabigatran and its effect on reaction time was determined. This relationship was explored to predict the target concentration (5 mg/L) known to be associated with a specific target effect (R-time 16 min). Initial dosing that could be used to study the drug during cardiopulmonary bypass was determined using estimated pharmacokinetic parameter estimates within a model that described a simple bypass circuit used in a sheep study. Subsequent monitoring of drug effect and/or drug concentrations and Bayesian forecasting may be used to improve the dose in individual sheep undergoing cardiopulmonary bypass.^17^

Monitoring of R-time is a convenient point-of-care whole blood test that is rapidly achieved. The short equilibration half-time (T_1/2_keo) makes this a coagulative measure useful for dabigatran dose adjustment. The need for subsequent monitoring after initiation of bypass is because the use of extracorporeal circuits are associated with additional covariates that can affect both pharmacokinetic (e.g., clearance, volume) and pharmacodynamic (E_MAX_, C_50_) parameter estimates. While it is possible to nonmaize pharmacokinetic parameters for size using allometric theory,^20,22,23^ pharmacokinetic maturation, particularly in children has impact.^22,24–26^ These considerations have been used to describe dabigatran in children suffering thromboembolic disease.^27^ However, these considerations becomes more complex in situations such as extracorporeal membrane oxygenation (ECMO) where improved organ perfusion betters function.^28^ In contrast, children undergoing cardiopulmonary bypass often suffer a renal insult, lessening clearance. Altered organ blood flow, temperature changes or inflammatory responses can affect clearance. Protein binding changes, haemodilution, and circuit drug adherence influence apparent volume of distribution. Haemodilution and inflammatory responses have further impact on the coagulation cascade. A lower target concentration during infancy may be required because of immaturity of the coagulation cascade.^29^

We have explored the sheep as an animal model because there is an increasing focus on rapidly translating findings in the laboratory to clinical applications in cardiovascular procedures. This has resulted in a shift from small to larger animal models, such as sheep, that closely correlate with human anatomy and physiology and allow use of commercially available bypass units used in humans. This current study was performed under general anaesthesia, a clinical scenario also used in humans. It is of value that the pharmacokinetic parameter estimate for clearance was similar to that noted in adult humans.^2,3^ Concerns that anaesthesia drugs may interfere with dabigatran metabolism are unfounded. Dabigatran in primarily cleared by renal function. Ketamine is metabolized by CYP2B6 and CYP3A4-mediated N -demethylation at a rate approximating hepatic blood flow.^30^ Midazolam is primarily metabolized by CYP3A4.^31^ These hepatic clearance pathways have little or no impact on renal function, glucuronide conjugation or dabigatran clearance.^32^ Isoflurane is predominantly cleared through the lungs rather than hepatic metabolism. The anaesthetic drugs are not known to have any coagulation effect. Neither renal function nor hepatic function were compromised in sheep given dabigatran.

The use of idarucizumab 15 mg/kg administered 120 min after dabigatran 4 mg/kg reduced reaction time by approximately 5 min over the 5 min after administration. An idarucizumab dose range was not explored. A larger dose may have greater effect. The reversal agent certainly had effect at this dose, reducing reaction time by 30%. The clinical impact of this effect on sheep while undergoing cardiopulmonary bypass remains untested.

## Supplemental Material

Supplemental Material - Dabigatran pharmacokinetic-pharmacodynamic in sheep: Informing dose for anticoagulation during cardiopulmonary bypass Supplemental Material for Dabigatran pharmacokinetic-pharmacodynamic in sheep: Informing dose for anticoagulation during cardiopulmonary bypass by Michael P Eaton, Sergiy M Nadtochiy, Tatsiana Stefanos and Brian J Anderson in Perfusion.

## Data Availability

The data that support the findings of this study are available from the corresponding author upon reasonable request.

## References

[1] Halton J Brandao LR Luciani M , et al. Dabigatran etexilate for the treatment of acute venous thromboembolism in children (DIVERSITY): a randomised, controlled, open-label, phase 2b/3, non-inferiority trial. Lancet Haematol 2021; 8: e22–e33. 33290737 10.1016/S2352-3026(20)30368-9

[2] Ganetsky M Babu KM Salhanick SD , et al. Dabigatran: review of pharmacology and management of bleeding complications of this novel oral anticoagulant. J Med Toxicol 2011; 7: 281–287. 21887485 10.1007/s13181-011-0178-y PMC3550194

[3] Stangier J Clemens A . Pharmacology, pharmacokinetics, and pharmacodynamics of dabigatran etexilate, an oral direct thrombin inhibitor. Clin Appl Thromb Hemost 2009; 15(Suppl 1): 9S–16S. 19696042 10.1177/1076029609343004

[4] Stangier J Rathgen K Stahle H , et al. Influence of renal impairment on the pharmacokinetics and pharmacodynamics of oral dabigatran etexilate: an open-label, parallel-group, single-centre study. Clin Pharmacokinet 2010; 49: 259–268. 20214409 10.2165/11318170-000000000-00000

[5] Glund S Coble K Gansser D , et al. Pharmacokinetics of idarucizumab and its target dabigatran in patients requiring urgent reversal of the anticoagulant effect of dabigatran. J Thromb Haemostasis 2019; 17: 1319–1328. 31050868 10.1111/jth.14476 PMC6852568

[6] Shazly A Afifi A . RE-ALIGN: first trial of novel oral anticoagulant in patients with mechanical heart valves - the search continues. Glob Cardiol Sci Pract 2014; 2014: 88–89. 25054124 10.5339/gcsp.2014.13 PMC4104382

[7] Koster A Yeter R Buz S , et al. Assessment of hemostatic activation during cardiopulmonary bypass for coronary artery bypass grafting with bivalirudin: results of a pilot study. J Thorac Cardiovasc Surg 2005; 129: 1391–1394. 15942583 10.1016/j.jtcvs.2004.09.016

[8] Koster A Spiess B Chew DP , et al. Effectiveness of bivalirudin as a replacement for heparin during cardiopulmonary bypass in patients undergoing coronary artery bypass grafting. Am J Cardiol 2004; 93: 356–359. 14759391 10.1016/j.amjcard.2003.10.021

[9] Wolstencroft P Arnold P Anderson BJ . Dose estimation for bivalirudin during pediatric cardiopulmonary bypass. Paediatr Anaesth 2021; 31: 637–643. 33423355 10.1111/pan.14125

[10] Nadtochiy SM Stefanos T Angona RE , et al. Rivaroxaban reduces the dabigatran dose required for anticoagulation during simulated cardiopulmonary bypass. Anesth Analg 2022; 135: 52–59. 35389372 10.1213/ANE.0000000000006019

[11] Nadtochiy SM Baldzizhar A Stefanos T , et al. High-dose dabigatran is an effective anticoagulant for simulated cardiopulmonary bypass using human blood. Anesth Analg 2021; 132: 566–574. 32833714 10.1213/ANE.0000000000005089

[12] Pollack CV Reilly PA Eikelboom J , et al. Idarucizumab for dabigatran reversal. N Engl J Med 2015; 373: 511–520. 26095746 10.1056/NEJMoa1502000

[13] Maas H Gropper S Huang F , et al. Anticoagulant effects of dabigatran in paediatric patients compared with adults: combined data from three paediatric clinical trials. Thromb Haemostasis 2018; 118: 1625–1636. 30112751 10.1055/s-0038-1668132 PMC6202931

[14] McMichael ABV Ryerson LM Ratano D , et al. 2021 ELSO adult and pediatric anticoagulation guidelines. Am Soc Artif Intern Organs J 2022; 68: 303–310. 10.1097/MAT.0000000000001652 35080509

[15] Hull CJ Van Beem HB McLeod K , et al. A pharmacodynamic model for pancuronium. Br J Anaesth 1978; 50: 1113–1123. 718781 10.1093/bja/50.11.1113

[16] Sheiner LB Stanski DR Vozeh S , et al. Simultaneous modeling of pharmacokinetics and pharmacodynamics: application to D-tubocurarine. Clin Pharmacol Ther 1979; 25: 358–371. 761446 10.1002/cpt1979253358

[17] Holford NHG . The target concentration approach to clinical drug development. Clin Pharmacokinet 1995; 29: 287–291. 8582116 10.2165/00003088-199529050-00001

[18] Holford NHG . Target concentration intervention: beyond Y2K. Br J Clin Pharmacol 2001; 52(Suppl 1): 55S–59S. 11564053 10.1046/j.1365-2125.2001.0520s1055.x PMC2014624

[19] Zhang L Beal SL Sheiner LB . Simultaneous vs. sequential analysis for population PK/PD data I: best-case performance. J Pharmacokinet Pharmacodyn 2003; 30: 387–404. 15000421 10.1023/b:jopa.0000012998.04442.1f

[20] West GB Brown JH Enquist BJ . A general model for the origin of allometric scaling laws in biology. Science 1997; 276: 122–126. 9082983 10.1126/science.276.5309.122

[21] Gonzalez-Sales M Holford N Bonnefois G , et al. Wide size dispersion and use of body composition and maturation improves the reliability of allometric exponent estimates. J Pharmacokinet Pharmacodyn 2022; 49: 151–165. 34609707 10.1007/s10928-021-09788-3

[22] Anderson BJ Holford NH . Mechanism-based concepts of size and maturity in pharmacokinetics. Annu Rev Pharmacol Toxicol 2008; 48: 303–332. 17914927 10.1146/annurev.pharmtox.48.113006.094708

[23] Anderson BJ Holford NH . What is the best size predictor for dose in the obese child? Pediatr Anesth 2017; 27: 1176–1184. 10.1111/pan.13272 29076211

[24] Holford N Heo YA Anderson B . A pharmacokinetic standard for babies and adults. J Pharm Sci 2013; 102: 2941–2952. 23650116 10.1002/jps.23574

[25] Peters JW Anderson BJ Simons SH , et al. Morphine pharmacokinetics during venoarterial extracorporeal membrane oxygenation in neonates. Intensive Care Med 2005; 31: 257–263. 15678314 10.1007/s00134-004-2545-5

[26] Anderson BJ Holford NH . Understanding dosing: children are small adults, neonates are immature children. Arch Dis Child 2013; 98: 737–744. 23832061 10.1136/archdischild-2013-303720

[27] Roshammar D Huang F Albisetti M , et al. Pharmacokinetic modeling and simulation support for age- and weight-adjusted dosing of dabigatran etexilate in children with venous thromboembolism. J Thromb Haemostasis 2021; 19: 1259–1270. 33636042 10.1111/jth.15277 PMC8251571

[28] Peters JW Anderson BJ Simons SH , et al. Morphine metabolite pharmacokinetics during venoarterial extra corporeal membrane oxygenation in neonates. Clin Pharmacokinet 2006; 45: 705–714. 16802851 10.2165/00003088-200645070-00005

[29] Eaton MP Alfieris GM Sweeney DM , et al. Pharmacokinetics of epsilon-aminocaproic acid in neonates undergoing cardiac surgery with cardiopulmonary bypass. Anesthesiology. 2015; 122: 1002–1009. 25723765 10.1097/ALN.0000000000000616 PMC4882606

[30] Herd DW Anderson BJ Holford NH . Modeling the norketamine metabolite in children and the implications for analgesia. Pediatr Anesth. 2007; 17: 831–840. 10.1111/j.1460-9592.2007.02257.x 17683400

[31] Anderson BJ Larsson P , et al. A maturation model for midazolam clearance. Pediatr Anesth. 2011; 21: 302–308. 10.1111/j.1460-9592.2010.03364.x 20704661

[32] Blech S Ebner T Lubwig-Schwellinger E , et al. The metabolism and disposition of the oral direct thrombin inhibitor, dabigatran, in humans. Drug Metab Dispos. 2008; 36: 386–399. 18006647 10.1124/dmd.107.019083

