# Supplementary material for: Dabigatran pharmacokinetic-pharmacodynamic in sheep: Informing dose for anticoagulation during cardiopulmonary bypass
Source: Perfusion. 2024 Jan 3;40(1):183–91. doi: 10.1177/02676591231226291 (PMC11715065; doi:10.1177/02676591231226291)
Supplement: Supplemental Material - Dabigatran pharmacokinetic-pharmacodynamic in sheep: Informing dose for anticoagulation during cardiopulmonary bypass [file sj-pdf-1-prf-10.1177_02676591231226291.pdf]

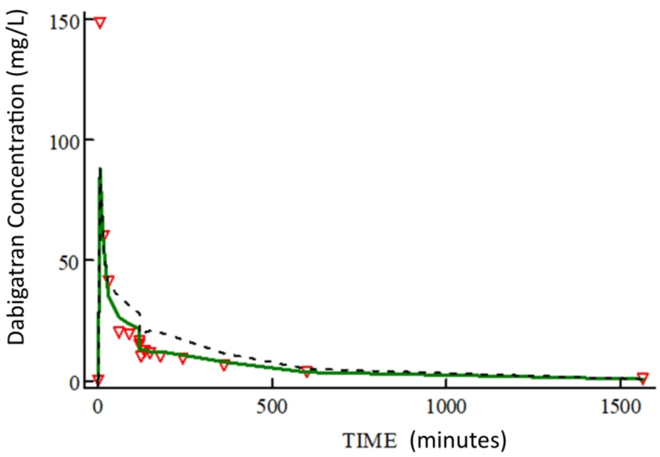

Supplementary Figure S1. Use of idarucizumab 15 mg/kg at 120 minutes after dabigatran 4 mg/kg lowered Reaction time by approximately 5 min. This figure demonstrates individual R-time observations (Δ). The individual Bayesian prediction is shown as a green line. Population prediction is a dashed line.

279x215mm (300 x 300 DPI)

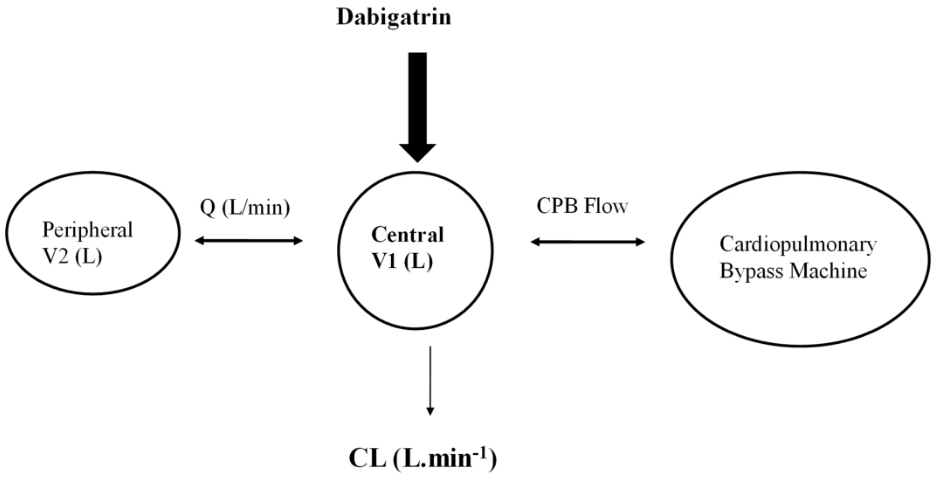

Supplementary Figure S2. A diagram representing the pharmacokinetic cardiopulmonary bypass model. Drug is delivered into a central compartment (V1) that distributes to a peripheral compartment (V2) linked by an intercompartment clearance (Q2). The cardiopulmonary bypass flow (Q3) connects cardiopulmonary machine to patient. The cardiopulmonary bypass machine has a known volume (V3)

230x173mm (300 x 300 DPI)

Supplementary NM-TRAN Code

```
$PROB dabigatran with IDA sheep basic deq

$INPUT ID TIME MDV DUR RATE AMT DVID DV WT CMT IDA

$DATA Sheep_dabi_IDA.csv IGNORE #

$ESTIM MAXEVAL=9999 NSIG=3 SIGL=9 PRINT=1 NOABORT METHOD=CONDITIONAL
INTERACTION

MSFO=dabi.msf

$COV

$THETA (0.01,0.0482,200); CL

$THETA (0.5,2.9,200)      ; V1

$THETA (0.001,0.269,200)    ; Q

$THETA (0.5,8.9,200)     ; V2

;RESIDUAL UNIDENTIFIED VARIABILITY (PK)

$THETA (0,1.3,)  ; RUV_SDCP

$THETA (0,0.0005,)  ; RUV_CVCP

$OMEGA BLOCK(4)

0.0602 ; PPVCL

-0.0112 0.00591 ; PPVV1

-0.0215 -0.0196 0.265 ;PPVQ

0.0285 -0.0292 0.153 0.165 FIX ; PPVV2

; PD

$THETA (100,180., 300 ) FIX    ; EMAX

$THETA (0.1,1.,8)    ; HILL

$THETA (0.5,64.2, 200)  ; C50

$THETA (0,0.681, 1)  ; E0

$THETA (0.001,1.04,200)   ; TEQ

; IDARUCIZUMAB

$THETA (0.001,0.0207,100)    ; KIDA

$THETA (0.01,0.0751,2)    ; POP_DURIDA

$THETA (0.001,8.68, 100)  ; SLOPE

;RESIDUAL UNIDENTIFIED VARIABILITY (PD)
```

```
$THETA (0,0.149,) ; RUV_SDPD
$THETA (0,0.412,) ; RUV_CVPD

$OMEGA BLOCK (2)
0.0395 ; PPVC50
0.0299 0.0227 ;PPVE0
$OMEGA 0 FIX                    ; PPVEMX
$OMEGA 0 FIX                    ; PPVTEQ
$OMEGA 0 FIX                    ; PPVHIL

$OMEGA 0 FIX                ; PPVDID
$OMEGA 1.03               ; PPVKID
$OMEGA 0 FIX                ; PPVSL

;RESIDUAL UNIDENTIFIED VARIABILITY (PD)
$OMEGA 0 FIX                    ; PPV_RUVCP
$OMEGA 0 FIX                    ; PPV_RUVPD
$SIGMA 1. FIX ; EPS1
$SUBR ADVAN6 TOL=5
$MODEL
 COMP (DIGAB)
 COMP (PERIPH)
 COMP (EFFECT)
 COMP (IDA)
$PK
IF (AMT.GT.0) DOSE=AMT
    IF (NEWIND.LE.1) THEN
        DOSE=0
        LN2=LOG(2)
   ENDIF
   FSZV=WT/70
   FSZCL=FSZV**0.75
```

```
 FSZT=FSZV**0.25

 CL=FSZCL*CL*EXP(PPVCL)
 Q=FSZCL*Q*EXP(PPVQ)
 V1=FSZV*V1*EXP(PPVV1)
 V2=FSZV*V2*EXP(PPVV2)

 E0=E0*EXP(PPVE0)
 C50=C50*EXP(PPVC50)
 EMAX=EMAX*EXP(PPVEMX)
 HILL=HILL*EXP(PPVHIL)
 TEQ=FSZT*TEQ*EXP(PPVTEQ)
 KEQ=LN2/TEQ

 SLOPE=SLOPE*EXP(PPVSL)
 DURIDA=POP_DURIDA*EXP(PPVDID)
 KIDA =KIDA*EXP(PPVKID)

 S1=V1
 S2=V2
 D1=DUR
 D4=DURIDA
$DES
 DCP=A(1)/V1
 DC2=A(2)/V2
 DCE=A(3)
 DIDA=A(4)

 DADT(1)= -DCP*CL-DCP*Q +DC2*Q
 DADT(2)= Q*DCP-DC2*Q
 DADT(3)= KEQ*(DCP-DCE)
 DADT(4)=-DIDA*KIDA
```

```
$ERROR
 CP=A(1)/V1
 CE=A(3)
 CIDA=A(4)

   "IF (CE.LE.0)CE=1D-10
  CEN=CE**HILL
  C50N=C50**HILL

 FX1=E0 + EMAX*CEN/(C50N+CEN)
 FX2=SLOPE*CIDA
 FX=FX1-FX2
 ;FX=E0*(1-EMAX/(1+(CE/C50)**(-HILL)))

   PROPP=CP*RUV_CVCP
   ADDP=RUV_SDCP
   SDCP=SQRT((PROPP*PROPP) + (ADDP*ADDP))*EXP(PPV_RUVCP)

   PROPD=CE*RUV_CVPD
   ADDPD=RUV_SDPD
   SDPD=SQRT((PROPD*PROPD) + (ADDPD*ADDPD))*EXP(PPV_RUVPD)

 IF(DVID.LE.1) THEN  ;; DIGABACTRAN CONCENTRATION
  Y=CP + SDCP*EPS1
 ENDIF

IF(DVID.EQ.2) THEN ;; EFFECT COAG
 Y=FX + SDPD*EPS1
ENDIF
$TABLE ID TIME WT CL V1 Q V2 Y CE MDV DVID WT
ONEHEADER NOPRINT FILE=dabi.fit
```
